# Supplementary material for: Microenvironmental cooperation promotes early spread and bistability of a Warburg-like phenotype
Source: Sci Rep. 2017 Jun 8;7:3103. doi: 10.1038/s41598-017-03342-3 (PMC5465218; doi:10.1038/s41598-017-03342-3)
Supplement: Supplementary file 1 — Supporting Text [file 41598_2017_3342_MOESM1_ESM.pdf]

SUPPORTING TEXT

Microenvironmental cooperation promotes early spread and bistability of a Warburg-like phenotype

Jorge Fernandez-de-Cossio-Diaz[1], Andrea De Martino[2,3,¶], and Roberto Mulet[4,¶]

[1]Department of Systems Biology, Center of Molecular Immunology, La Habana (Cuba)
[2]Soft and Living Matter Lab, Istituto di Nanotecnologia (CNR-NANOTEC), Rome (Italy)
[3]Human Genetics Foundation, Turin (Italy)
[4]Group of Complex Systems and Statistical Physics, Department of Theoretical Physics, Physics Faculty, University of Havana (Cuba)
[¶]*Co-last authors*

Mathematical analysis of the homogeneous model

The Michaelis-Menten equations for the primary nutrient intake by aberrant and healthy cells, i.e.

$$u_{\text{ab}} = \alpha_{\text{ab}} u_{\max} \frac{s}{s + K_{\text{s}}} \tag{1}$$

$$u_{\text{he}} = u_{\max} \frac{s}{s + K_{\text{s}}} \tag{2}$$

can be inverted to obtain the concentration s consistent with given uptake fluxes. For aberrant cells one gets

$$s = \frac{K_{\text{s}} u_{\text{ab}}}{\alpha_{\text{ab}} u_{\max} - u_{\text{ab}}} \tag{3}$$

The thresholds s_{sec} and s_{gr} (see Eq. (13) in the Main Text) are obtained by replacing u_{ab} with $r_{\max}$ and u_m respectively. Similarly, for healthy cells under nutrient limitation, one has

$$s = \frac{K_{\text{s}} u_{\text{he}}}{u_{\max} - u_{\text{he}}} \tag{4}$$

and s_m is derived by replacing u_{he} with u_m. Note that, in both cases, s is a decreasing function of $u_{\max}$, which proves that $s_m > s_{\text{gr}}$ (assuming $\alpha_{\text{ab}} > 1$). Also, s is increasing in both u_{ab} and u_{he}, implying that $s_{\text{sec}} \geq s_{\text{gr}}$ (assuming that $r_{\max} \geq u_m$). Finally, by straightforward algebraic manipulation, the inequality $s_m > s_{\text{sec}}$ can be shown to correspond to $u_m \alpha_{\text{ab}} > r_{\max}$, the necessary condition for the occurrence of the waste shuttle discussed in the Main Text.

By the quasi-steady state approximation for the dynamics of s and w, we have

$$-\phi u_{\text{ab}} - (1-\phi) u_{\text{he}} + k(c - s) = 0 \tag{5}$$

$$-\phi v_{\text{ab}} - (1-\phi) v_{\text{he}} - kw = 0 \tag{6}$$

which can be solved for s and w as functions of ϕ. In turn, one obtains an expression for ϕ as a function of s, namely

$$\phi = \frac{F(c - s) - u_{\text{he}}}{u_{\text{ab}} - u_{\text{he}}} \tag{7}$$

where u_{he} and u_{ab} are Michaelis-Menten functions of s. Upon substituting these, one can show that ϕ decreases as s increases (and vice-versa). Moreover, replacing s with each of the thresholds in Eq. (13) of the Main Text one obtains the matching thresholds for ϕ given in the Main Text as Eq. (14).

Non-dimensional parameters

The variables and parameters of the model are summarized in Table 1, along with their physical units. Then we define non-dimensional quantities summarized in Table 2.

Symbol	Meaning	Units
Variables		
ϕ	Fraction of aberrant cells	-
s	Extracellular glucose concentration	molecules / volume
w	Extracellular lactate concentration	molecules / volume
Metabolic fluxes		
u_{he}	Glucose uptake of normal cells	concentration / time
u_{ab}	Glucose uptake of aberrant cells	concentration / time
v_{he}	Lactate uptake of normal cells	concentration / time
v_{ab}	Lactate uptake of aberrant cells	concentration / time
e	ATP production rate	concentration / time
Michaelis-Menten constants		
u_{max}	Maximum glucose uptake of normal cells	concentration / time
K_{s}	Glucose uptake Michaelis constant	molecules / volume
u_{max}	Maximum lactate uptake of normal cells	concentration / time
K_{w}	Lactate uptake Michaelis constant	molecules / volume
Replication and death rates		
λ	Replication rate	1 / time
σ	Death rate	1 / time
Replication and death related constants		
λ_{max}	Maximum replication rate	1 / time
σ_{max}	Maximum death rate	1 / time
K_{E}	Replication rate half-saturation	concentration / time
K_{inh}	Replication inhibition half-saturation	molecules / volume
K_{tox}	Death rate half-saturation	molecules / volume
Spatial constants		
$D_{\mathrm{S}}, D_{\mathrm{W}}$	Diffusion coefficients	1 / time
h	Linear dimension of cells	distance
External parameters		
k	Turn-over rate	1 / time
c	Blood glucose concentration	molecules / volume

Table 1. List of model parameters with their respective units.

Dimensional quantity	Non-dimensional form
Concentrations	
s	$\frac{2s}{K_{\text{tox}}}$
w	$\frac{w}{K_{\text{tox}}}$
Metabolism	
u	$\frac{2u}{K_{\text{tox}}\sigma_{\max}}$
v	$\frac{v}{K_{\text{tox}}\sigma_{\max}}$
e	$\frac{e}{K_{\text{tox}}\sigma_{\max}}$
$u_{\max}$	$\frac{2u_{\max}}{K_{\text{tox}}\sigma_{\max}}$
$v_{\max}$	$\frac{v_{\max}}{K_{\text{tox}}\sigma_{\max}}$
K_{s}	$\frac{2K_{\text{s}}}{K_{\text{tox}}}$
K_{w}	$\frac{K_{\text{w}}}{K_{\text{tox}}}$
e_m	$\frac{e_m}{K_{\text{tox}}\sigma_{\max}}$
u_m	$\frac{u_m}{K_{\text{tox}}\sigma_{\max}}$
Replication and death related parameters	
λ	$\frac{\lambda}{\sigma_{\max}}$
σ	$\frac{\sigma}{\sigma_{\max}}$
K_{E}	$\frac{K_{\text{E}}}{K_{\text{tox}}}$
K_{inh}	$\frac{K_{\text{inh}}}{K_{\text{tox}}}$
K_{tox}	1
$\lambda_{\max}$	$\frac{\lambda_{\max}}{\sigma_{\max}}$
$\sigma_{\max}$	1
Time	
t	$\frac{t}{\sigma_{\max}}$

Table 2. Definitions of nondimensional parameters.
